# Factor structure of the Shoulder Pain and Disability Index in patients with adhesive capsulitis

**DOI:** 10.1186/1471-2474-9-103

**Published:** 2008-07-17

**Authors:** Einar Kristian Tveitå, Leiv Sandvik, Ole Marius Ekeberg, Niels Gunnar Juel, Erik Bautz-Holter

**Affiliations:** 1 Department of Physical Medicine and Rehabilitation, Ullevål University Hospital, University of Oslo, Oslo, Norway; 2 Department of Biostatistics and Epidemiology, Ullevål University Hospital, University of Oslo, Oslo, Norway

## Abstract

**Background:**

The Shoulder Pain and Disability Index (SPADI) is a self-administered questionnaire that aims to measure pain and disability associated with shoulder disease. It consists of a pain section and a disability section with 13 items being responded to on visual analogue scales. Few researchers have investigated SPADI validity in specified diagnostic groups, although the selection of an evaluative instrument should be based on evidence of validity in the target patient group. The aim of the present study was to investigate factor structure of the SPADI in a study population of patients with adhesive capsulitis.

**Methods:**

The questionnaire was administered to 191 patients with adhesive capsulitis. Descriptive statistics for items and a comparison of scores for the two subscales were produced. Internal consistency was analyzed by use of the Cronbach alpha and a principal components analysis with varimax rotation was conducted. Study design was cross-sectional.

**Results:**

Two factors were extracted, but the factor structure failed to support the original division of items into separate pain and disability sections.

**Conclusion:**

We found minimal evidence to justify the use of separate subscales for pain and disability. It is our impression that the SPADI should be viewed as essentially unidimensional in patients with adhesive capsulitis.

## Background

The Shoulder Pain and Disability Index (SPADI) is a self-administered questionnaire, designed by Roach and colleagues to measure the impact of shoulder pathology in terms of pain and disability, for both current status and change over time [1]. The questionnaire consists of 13 items grouped into pain and disability subscales, the questions starting with "How severe is your pain...?" and "How much difficulty do you have...?", respectively. Items mainly deal with various activities of daily living (ADL) that may or may not be problematic to the patient. Items are rated on visual analogue scales to produce a score for each subscale, and the means of the two subscales are averaged to produce a total score ranging from 0 (best) to 100 (worst).

While responses to individual items are observable and concrete variables, the concepts they purport to assess when combined are abstract or latent variables, socalled "constructs" [2]. For a meaningful comparison of scores, it is essential that scores reflect the same construct. To accommodate the interpretation of scores from multi-item questionnaires, health assessment scales are often divided, as is SPADI, into subscales with presumably separate constructs. In several cases, subsequent research has, however, failed to support the original structure of such scales [3-5], demonstrating uncertainty regarding scale appropriateness in many settings.

SPADI is one of the shoulder rating scales that has been most extensively studied [3,6]. Construct validity (the extent to which a measure assesses the domain of interest [2]) of the SPADI was investigated by the original developers through factor analysis. This is a statistical technique applied to a group of items to determine if the items form coherent subsets that are relatively independent from one another [2]. In the beginning of the original SPADI article, the authors hypothesized that "Varimax rotation should produce two factors with the majority of items from each subscale primarily loading on different factors". However, the factors extracted did not delineate clearly between items of the pain and disability subscales in that original study, perhaps because the study was undersized [1]. Three subsequent studies have come to diverging conclusions regarding SPADI factor structure. Only one factor was retained in studies by Roddey et al. [7] and Placzek et al. [4], but the original division of the questionnaire was supported in a study [8] by MacDermid and colleagues.

The selection of an evaluative instrument should be based on evidence of reliability and validity in the target patient population [9]. It has been argued that a shoulder rating scale should be equally valid across diagnostic categories. This may be far-fetched within the format of instruments like SPADI. Patients with different shoulder conditions may have functional limitations that need to be addressed in separate ways. Certain patient groups are mostly inhibited by pain while others experience e.g. limited strength or flexibility. Some conditions only become a problem when performing specific and highly demanding activities like throwing or weight-lifting.

Adhesive capsulitis is one of the most common disorders affecting the shoulder and SPADI has been employed in several clinical trials involving this patient category [10-14]. The main findings associated with active adhesive capsulitis is a constricted glenohumeral joint capsule and a shoulder pain that becomes severe if the arm is passively or actively moved towards limits of range-of-motion. Symptoms indicate that both pain and disability parameters may apply when characterizing patients. SPADI items seem to address constructs of pain or disability in various situations, and in this sense, face validity is promising. However, it is uncertain whether the two can be assessed separately in this way since pain and disability may be very closely connected in these patients. The aim of the present study is to investigate if the underlying factor structure of the SPADI supports a separation of scores into different subscales when describing patients with adhesive capsulitis.

## Methods

### Participants

The study was conducted as part of a larger project regarding adhesive capsulitis and outcome measures for this condition. The Regional Ethics Committee for Eastern Norway granted ethical approval for the project. We prepared a Norwegian version [15] of the SPADI by translation according to internationally accepted guidelines [16]. The Norwegian version was routinely administered to shoulder patients as they showed up for appointments at our outpatient clinic in the period May 2003 to February 2006. Study design was cross-sectional and questionnaires were responded to anonymously. Results in this study are based on scores from a total of 191 patients with adhesive capsulitis who filled the following criteria: 1) limitation of passive movement in the glenohumeral joint of more than 30 degrees for at least two of these three movements: forward flexion, abduction or external rotation [10] and 2) willingness and ability to fill out the SPADI. The range-of-motion criterium was used in order to obtain a study population that would resemble study populations in relevant trials where the SPADI has been employed as an outcome measure. Data regarding age, sex and duration of the condition were registered along with SPADI scores. Some of the patients were included in a separate study investigating treatment effects of hydrodilatation and corticosteroid injections.

### Measurements

SPADI is a self-administered instrument aiming to measure pain and disability associated with shoulder disease. It consists of five pain and eight disability items. Each item is measured on an 11 cm visual analogue scale, producing figures ranging from 0 to 10. Pain and disability subscale scores are calculated as the mean of the corresponding items on a 0–100 scale, the highest score indicating the most severe pain and disability. The total score is calculated as the average of the pain and disability subscales. If more than two items of a subscale are not responded to, no SPADI score is calculated [1].

### Statistical procedures

Descriptive statistics were calculated for the 13 items, the subscales and the total score. Inter-subscale correlation was examined with the Pearson correlation coefficient (*r*). Agreement between the subscales was investigated with Bland-Altman scatterplots [17]. Cronbach alpha was calculated for the subscales and for the SPADI total [2,18].

In a factor analysis, the factor solution is based on the co-variances that arise from the relationship of items to underlying latent variables, known as common factors. The common factors are considered to represent underlying concepts, and factor analyses can be used to test the construct validity of a measure [2]. In this study, items were analyzed by a principal components factor analysis [19]. The Kaiser-Meyer-Olkin Measure and Bartlett's Test of Sphericity [20] were computed to determine whether the data were suitable for factor analysis. Initial factors were extracted according to the Kaiser criterion of retaining eigenvalues larger than 1.00. Factor eigenvalues reflect the amount of variance accounted for by each factor. The use of the eigenvalue larger than 1.00 criterion is equivalent to setting the minimum variance explained at the (100/number of items) percent level [19]. A varimax rotation method was used to obtain independent factors and an item was considered to be loaded on a factor if the matrix coefficient was 0.50 or larger [20].

All statistical analyses were carried out using the software package SPSS 13.0 for Windows^® ^(SPSS, Chicago, IL, USA) and the recommendations by Andy Field [20].

## Results

### Descriptives

**Figure 1:**
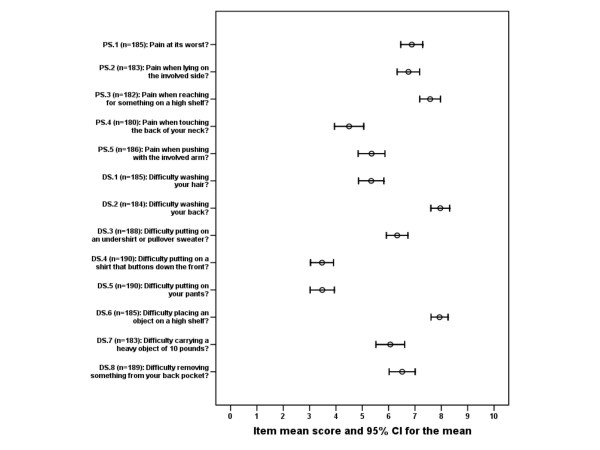
**Item mean scores**. Mean scores for each item with 95% confidence intervals (CI) for the means.

Mean age of the participants was 51.9 years (SD 8), 111 (58%) were female, and median duration of the condition was 7.0 months. 148 (77%) out of the 191 patients responded to all items in the questionnaire. The numbers of respondents for each item with mean scores and 95% confidence intervals for the means are given in Figure 1. There is considerable inter-item variability in mean score, indicating a "hierarchy" regarding item difficulty. The mean score for the pain subscale was 60.7 (SD 22) on the 0 – 100 scale, mean score for the disability subscale was 58.1 (SD 20) and mean total score was 59.4 (SD 19).

### Internal consistency

**Figure 2:**
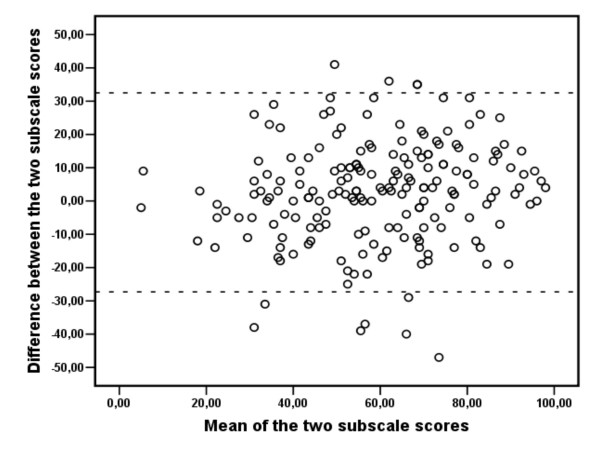
**Bland-Altman scatterplot of agreement between subscales**. Mean of the two subscales plotted against the difference between subscale scores for each patient (n = 191). Dotted lines indicate "limits of agreement" (mean difference +/- 1.96 SD of score difference).

Correlation between the subscales was 0.73. Agreement between the subscale scores is visualized in a Bland-Altman scatterplot (Figure 2). The difference between scores for each subscale is less than approximately 30 points for 95% of the respondents. Cronbach alpha values were 0.80 for the pain subscale, 0.87 for the disability subscale and 0.90 for the SPADI total.

### Principal components analysis

**Figure 3:**
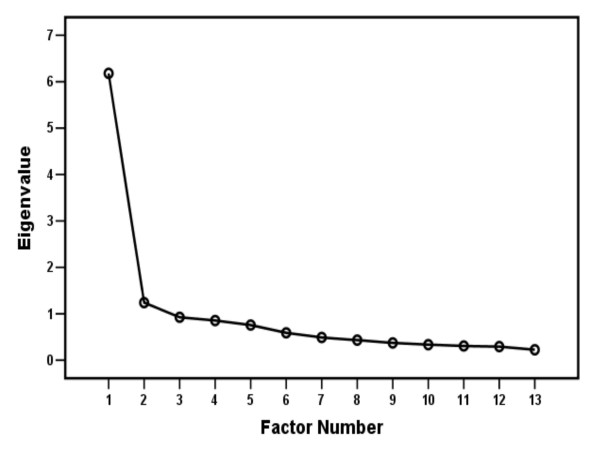
**Scree plot**. Each factor in the unrotated solution plotted against its eigenvalue (n = 148).

Results are based on the 148 patients who responded to all items in the questionnaire. The data met the Kaiser-Meyer-Olkin Measure and Bartlett's Test of Sphericity criteria for factor analysis. A one-factor solution explained 48%, a two-factor solution 57% and a three-factor solution 64% of the total variance. Using the eigenvalue criterion, two factors were extracted. Eigenvalues of initial factors are given in a scree plot [21] in Figure 3.

The total proportions of variance for each item explained by the two extracted factors are given in Table 1 in the column "Communalities".

**Table 1:** Communalities and factor loadings for individual items

**Items**	**Communalities**	**Factor 1**	**Factor 2**
	**(both factors)**	**loading**	**loading**
Pain section:			
PS.1 At its worst?	0.50	**0.67**	0.23
PS.2 When lying on the involved side?	0.57	**0.66**	0.36
PS.3 Reaching for something on a high shelf?	0.66	**0.80**	0.16
PS.4 Touching the back of your neck?	0.41	0.44	0.46
PS.5 Pushing with the involved arm?	0.48	0.44	**0.54**
			
Disability section:			
DS.1 Washing your hair?	0.64	**0.56**	**0.58**
DS.2 Washing your back?	0.61	**0.76**	0.19
DS.3 Putting on an undershirt or pullover sweater?	0.50	0.41	**0.58**
DS.4 Putting on a shirt that buttons down the front?	0.71	-0.04	**0.84**
DS.5 Putting on your pants?	0.71	0.27	**0.80**
DS.6 Placing an object on a high shelf?	0.68	**0.81**	0.15
DS.7 Carrying a heavy object of 10 pounds?	0.47	0.49	0.48
DS.8 Removing something from your back pocket?	0.47	**0.57**	0.38

Items considered to load on a factor are bolded.

**Figure 4:**
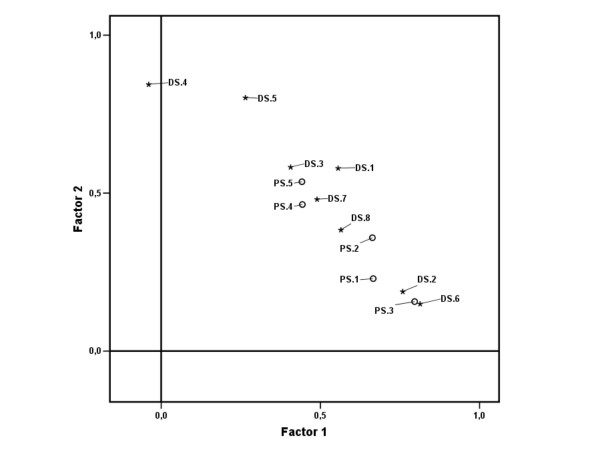
**Factor plot**. Loading on factor 1 plotted against loading on factor 2, for each item. Markers indicate pain section (PS) items numbered 1–5 and disability section (DS) items numbered 1–8, see Figure 1. Varimax rotation method (n = 148).

According to the rotated two-factor solution, 33% of the total variance was explained by the first factor and 24% by the second factor. Individual item loadings for these factors are given in Table 1. Three pain items and four disability items loaded on the first factor with a coefficient above 0.50, while one pain item and four disability items loaded on the second factor. One item loaded on both factors. Items loading on the first factor generally have higher mean scores than items loading on the second factor (compare Figure 1 and Table 1 for the items DS.4/DS.5 and PS.3/DS.2/DS.6). A graphical representation of the factor loadings for each item is given in Figure 4[20].

## Discussion

The factor structure identified in this study does not support the original division of the SPADI since the two extracted factors do not seem to delineate clearly between pain and disability subscales. The questionnaire clearly asks the patient to report pain in the first section and difficulty in the last, but it is unclear if the difference is appreciated by the patients. The factor structure revealed in this study is in line with previous reports on the construct validity of the SPADI.

Region-specific scales used in rheumatology or orthopedics tend to include items that refer to pain and various aspects of limited functioning associated with activities of daily living. For some scales, the association between pain and function has been shown to be weak, while for others it seems to be stronger [4]. It has been proposed that patients in some cases may have difficulties in separating the concepts because activities are essentially limited by pain [5]. Others have proposed that pain and disability items in questionnaires may correlate because pain and disability items tend to address similar tasks [22].

Critical to the analysis of the factor structure of a scale is deciding the number of factors to extract before rotation. In this study, two initial factors were extracted according to the eigenvalue criterion, a result that would seem to fit the number of constructs addressed in SPADI.

From a biomechanical perspective, it is tempting to label the first factor in the rotated solution "Pain interference" and the second factor "Functional limitation". Patients with adhesive capsulitis in the active stage experience an aggravation of pain when the arm is moved towards the limits of range-of-motion. The disability items that load on the first factor involve movements near (or beyond) end-range of shoulder motion in these patients. Hence it is not surprising that some "difficult" items in the disability section may load on pain. The interpretation would be that disability subscale scores depend on both pain interference and functional limitation.

The more pragmatic researcher might prefer to view both factors as "Pain interference" factors, the difference between them being pain interference with higher and lower demand activities, respectively. Variability in item differentiation may reflect clinical phenomena, but interpretational and psychological issues may be as relevant. Considerations regarding scoring procedures also apply.

Internal consistency was slightly lower than reported by previous researchers. Cronbach alpha was 0,90 for the total score, compared to Roach 0.95 [1], Roddey 0.96 [7], MacDermid 0.95 [8] and Angst 0.95 [23]. The value still indicates that the scale may be viewed as unidimensional.

Inter-subscale correlation (0.73) was in line with previous findings (Roach 0.87 [1], Placzek 0.71 [4], Roddey 0.77 [7], MacDermid 0.66 [8]). Investigation of agreement between the pain and disability subscales indicated that scores could vary by as much as 30 points for an individual patient, although the difference on average was only 3 points. A 30-point difference of this type is more than you would expect in a simple test-retest study of each subscale, indicating that subscale scores are not interchangeable for all patients.

The results of this study largely conform with the reports of Roach et al. [1], Placzek et al. [4] and Roddey et al. [7], in the sense that we have identified a factor loading pattern that does not support the original subscale division of items. This result may appear to contrast with the factor structure reported in the study by MacDermid et al., where one factor loaded primarily on pain items and another factor primarily on disability items [8]. However, the authors noted that higher demand activities tended to load on pain instead of disability. In MacDermid's study of community volunteers who self-identified as having shoulder pain, mean score of the disability section was 32. In our study it was 58. We find it reasonable that the "disability" items reflect pain interference to a higher degree in our study population of patients with adhesive capsulitis.

## Conclusion

It is our impression that the SPADI should be viewed as essentially unidimensional in this study population of shoulder capsulitis outpatients. Patients consider pain to be an essential part of what makes shoulder-related activities difficult, and as a consequence subscale scores tend to reflect the same construct.

## Competing interests

The authors declare that they have no competing interests.

## Authors' contributions

EKT, NGJ and EB–H designed the study. EKT, OME, NGJ and EB–H collected the data. EKT and LS performed the statistical analysis. EKT drafted the manuscript with contributions from LS, OME, NGJ and EB–H. All authors read and approved the final manuscript.

## Pre-publication history

The pre-publication history for this paper can be accessed here:


